# Evaluation the Presence of SERPINA5 (Exon 3) and FTO rs9939609 Polymorphisms in Papillary Thyroid Cancer Patients

**DOI:** 10.31557/APJCP.2021.22.11.3641

**Published:** 2021-11

**Authors:** Seyed Mohammad Moshtaghioun, Nasim Fazel-Yazdi, Mohammad Mandegari, Ahmad Shirinzadeh-Dastgiri, Mohammad Vakili, Habib Fazel-Yazdi

**Affiliations:** 1 *Department of Biology, Faculty of Science, Yazd University, Yazd, Iran. *; 2 *Department of Otolaryngology, Head and Neck Surgery, Otorhinolaryngology Research Center, Shahid Sadoughi University of Medical Sciences, Yazd, Iran.*; 3 *Department of Surgery, School of Medicine, Shohadaye Hafte-e-tie hospital, Iran University of Medical Sciences, Tehran, Iran. *; 4 *Department of Surgery, School of Medicine, Ardabil University of Medical Sciences, Ardabil, Iran.*; 5 *Yazd Diabetes Research Center, Shahid Sadoughi University of Medical Sciences, Yazd, Iran. *

**Keywords:** Papillary thyroid cancer, SERPINA5, FTO, PCR-SSCP, variation

## Abstract

**Background:**

A few researches evaluated the association of polymorphisms at SERPINA5 and fat mass and obesity-associated protein (FTO) genes with papillary thyroid cancer (PTC) globally. Here, we examined the presence of genetic variations within coding exon 3 of SERPINA5 gene and FTO rs9939609 polymorphism in Iranian PTC patients.

**Methods:**

A total of 122 patients (42 cases for SERPINA5 and 80 cases for FTO gene) and 120 healthy subjects (40 subjects or SERPINA5 and 80 subjects for FTO gene) were recruited. The genetic variation within coding exon 3 of SERPINA5 gene was evaluated by reaction-single-strand conformation polymorphism (PCR-SSCP) and FTO rs9939609 polymorphism was evaluated by RFLP-PCR assay.

**Results:**

The PCR-SSCP technique detected two rs6115G>A and rs6112T>C genetic variations within coding exon 3 of SERPINA5 gene and approved also by direct sequencing. For rs6112T>C polymorphism seven patients was heterozygous and for rs6115G>A seven PTC patients were heterozygous and two patients were homozygous.

**Conclusion:**

This study indicated that SERPINA5 rs6115G>A and rs6112T>C polymorphisms might be a novel susceptibility locus for PTC in Iranian patients. However, our findings do not support an association between FTO rs9939609 polymorphism and PTC risk.

## Introduction

Cancer is a leading cause of death globally, accounting for nearly 10 million deaths yearly (Mousavi et al., 2008; Behtash et al., 2009; Zarchi et al., 2010; Bineshet al., 2012; Khoram-Abadiet al., 2016). The worldwide incidence of thyroid cancer (TC) is increasing and accounts for some 10% of total cancer incidence (Shiryazdiet al., 2014; Jafari-Nedooshanet al., 2017). The frequency of this carcinoma is observed three times more in women than men. Thyroid neoplasms are classified histologically into tumors derived from follicular cells (papillary, follicular, and anaplastic carcinoma) and parafollicular or C cells (medullary carcinoma) (Valizadehet al., 2020; Farajkhodaet al., 2021; Phamet al., 2021). Papillary thyroid carcinoma (PTC) is the most common (80%) and has the highest cure rate in patients. Despite the notable increasing incidence of PTC in recent years, its optimal treatment remains controversial (La Vecchiaet al., 2015; Abbasiet al., 2020; Gohariet al., 2020).

To date, a large effort to investigate the genetic component of PTC has been performed (Hińczaet al., 2019; Ahmadiet al., 2021). SERPINA5 gene codes the protein C inhibitor, a member of the plasma serine protease inhibitor family, which known to play a role in many biological processes beyond hemostasis including inflammation, innate immunity, fertilization and carcinogenesis (Brenneret al., 2013; Sigurdsonet al., 2016). To date, a few studies evaluated the role of SERPINA5 gene in initiation and progression of malignancies. A study showed that expression of SERPINA5 in tissue of PTC patients was decreased and associated with the presence of BRAF mutation, believed to be one of the major drivers of thyroid carcinogenesis. In other study they reported that a strong associations of several SERPINA5 polymorphisms with PTC susceptibility among US-American patients (Kebebewet al., 2007; Lee et al., 2013). In other study, Selvam et al., reported that dysregulation of key proteins associated with sperm fertility potential and motility such as SERPINA5 in the cancer group. Moreover, it is reported that expression of the MEK-ERK pathway inhibitory gene SERPINA5, which is regulated by the methylation of its DNA promoter (Selvamet al., 2019, 2020). The overexpression of DUSP4 and SERPINA5 was associated with the indolent behavior of BRAF-positive ovarian tumors due to suppression of matrix metalloproteinase (MMP9) activity (Leeet al., 2013; Dastgheibet al., 2016). Moreover, some studies suggested that polymorphisms at fat mass and obesity associated (FTO) gene might be significantly associated with PTC risk, while the results of some other studies were controversial. Thus, we carried out this study to evaluate the association of the association of genetic variations within coding exon 3 of SERPINA5 and rs9939609 polymorphism with susceptibility to PTC in Iranian patients.

## Materials and Methods

*Participants*

Ethics approval of this study was issued by the Ethics Committee of Yazd University (IR.YU.SPH.REC.1398.098), Yazd, Iran. All procedures in the current study were performed in accordance with the ethical standards of the institutional or national research committee and with the 1964 Declaration of Helsinki and its later amendments or comparable ethical standards. The objective of the study was fully explained to all participants and a written informed consent was obtained from them. This study was approved by the Ethics Committee of the Yazd University. The eligible participants corresponded to a total of 122 patients (42 cases for SERPINA5 and 80 cases for FTO gene) and 120 healthy subjects (40 subjects or SERPINA5 and 80 subjects for FTO gene) without any history of malignancy considered as the control group. All of PTC patients were histologically confirmed by pathologists. The PTC case and healthy subjects were gender and age matched.

*DNA extraction and Genotyping*

*SERPINA5 Exon 3*

Genomic DNA was extracted from 200 μL of peripheral blood of all subjects with EDTA as an anticoagulant. A Commercially available kit for DNA extraction was used for DNA extraction, according to the manufacturer’s instructions kit (purchased from GeneAll Co., LTD). The SERPINA5 polymorphisms were genotyped using Single strand conformation polymorphism (SSCP) technique. Polymerase chain reaction (PCR) amplification of genomic DNA was performed using one pair of primer, scanning the coding exon 3 of SERPINA5 gene. The primer sequences and product size have listed in Table 1. PCR amplification was performed in a final volume of 25 μl containing 100 ng total DNA as a template, 0.5 μl each primers (10 pmol), 0.8 μl MgCl2, 0.5 μl of dNTP, 2.5 μl of 10X PCR buffer and 2 Units Taq DNA polymerase. The PCR was performed based on the following conditions: initial denaturation at 95°C for 5 min; followed by 30 cycles including denaturation at 95°C for 30 s, annealing at 61.7°C for 50 s, and extension at 72°C for 35s; and a final extension at 72°C for 5 min. The PCR products were electrophoresed on 1.5% agarose gel and stained with ethidium bromide. Then, PCR-SSCP assay was used in order to screening the mutations. For this analysis, 5μl of PCR products were mixed with 2μl of SSCP loading dye. The samples were denatured at 94°C for 10min and cooled on ice. The fragments showed abnormal migration to elucidate the type of mutation, samples with different band were sent for directly sequencing. The control samples were selected from 40 unrelated, ethnically normal controls.

*FTO rs9939609 Polymorphism*

Genomic DNA was extracted by DNA isolation kit according to the manufacturer’s recommendations. The DNA quantity was measured NanoDrop (ND1000, USA), and the extracted DNA was stored at -20°C until use. The FTO rs9939609 polymorphism was genotyped using RFLP- PCR. The primer sequences and product size have listed in Table 1. The PCR reaction in of total volume 20 μl was performed with a mixture containing 5µl genomic DNA, 5µl dNTPs, 1µl of each primer and 0.5µl Taq DNA polymerase which was added into 3µl of PCR buffer containing 10 mMTris-HCl, 1.5 μM MgCl_2_ and 50 mMKCl and added the double distilled water to make up the total volume. The amplification step consisted of 40 cycles of 45 s at 95°C, 60 s at 59°C, in addition to 72°C for 45 s with a final extension of 5 min at 72°C. The product was digested with 5ul (10x) ScaI restriction endonucleases at 37˚C for 8 hours. Then, the products add were loaded on a buffer and run on 1.5% Agarose gel for 1 hour. The product sizes were as: a band of 187 bp for the TT genotype, two bands of 154, 33 bp for the AA genotype and three bands of 187, 154, and 33 bp for AT heterozygous genotype.

*Statistical Analysis*

The chi-square test or Fisher’s exact test was performed to examine the differences between cases and controls in terms of mean age and gender. Hardy-Weinberg equilibrium (HWE) was used for the distributions of FTO rs9939609 polymorphism in healthy subjects performed by the chi square (*χ*^2^) test. The associations of FTO rs9939609 polymorphism with PTC risk was calculated by using the odds ratio (OR) and 95% confidence interval (CI) (Jafari-Nedooshanet al., 2019; Bahrami et al., 2020; Bahrami et al., 2020; Jarahzadehet al., 2021). Statistical analyses were carried out using SPSS version 19.0 (SPSS Co., Chicago, IL, USA) for Windows.

## Results

*SERPINA5 exon 3*

**Figure 1:**
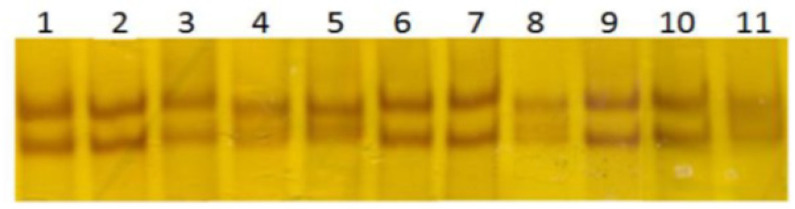
Agarose Gel Electrophoresis (1.5%) of PCR-SSCP Product for Exon3 of SERPINA5 Gene. Lanes 8-11 represents unrelated healthy controls, lanes 4 and 5 represents heterozygous- mutant, lanes 1,2,3,6 and 7 represents patients with similar banding pattern to unrelated healthy controls

**Figure 2:**
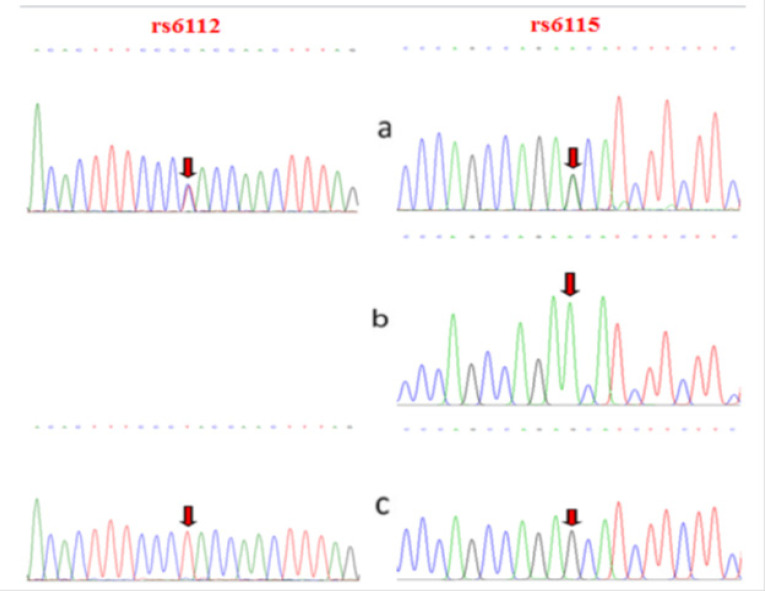
Direct DNA Sequence Analysis Results. a, Sequence electropherogram for a heterozygous-mutant; b, Sequence electropherogram for a homozygous-mutant; c, Sequence electropherogram from homozygous-wild type. The arrow shows the site of mutation

**Figure 3:**
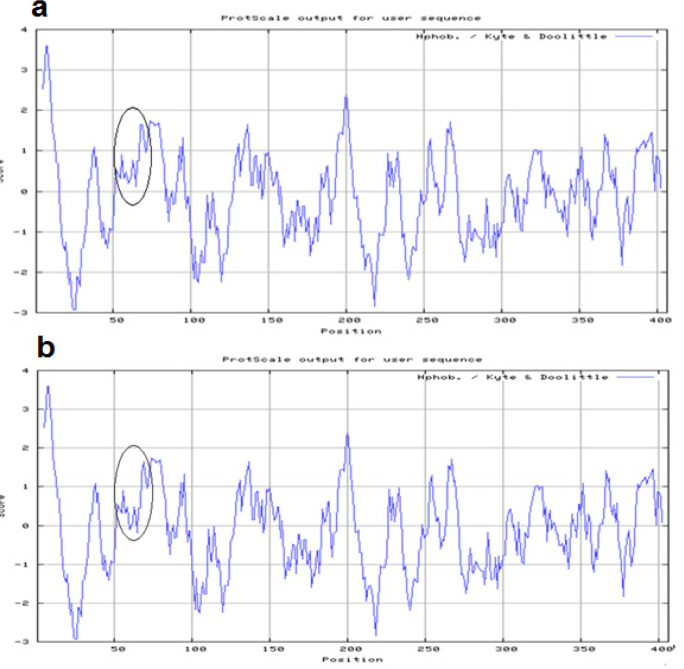
Comparison of Normal (a) and Mutant Protein Hydrophobicity (b) Caused by SERPINA5 rs6115G>A Polymorphism

Exons 3 of the SERPINA5 gene were screened for the presence of mutations by PCR SSCP. As shown in Figures 1 and 2, DNA fragments showed abnormal banding patterns on SSCP analyses were sequenced for the identification of exact variation. The analyses identified two rs6115 and rs6112 genetic variations within coding exon 3 of SERPINA5 gene and approved also by direct sequencing. For rs6112 T>C polymorphism seven patients was heterozygous and for rs6115 G>A seven PTC patients were heterozygous and in two patients were homozygous. These nucleotide variations have been observed simultaneously in five patients. The rs6112 T>C was not caused an amino acid change at position 159 (Proline to Proline; Pro> Pro). However, rs6115 G>A was associated with an amino acid changes at position 64 (serine to asparagine; Ser>Asn). Those two amino acids changes caused by rs6115G>A are two uncharged polar (hydrophilic), therefore, protein hydrophobicity significantly doesn’t change (Figure 3). But, the rs6115G>A polymorphism may affect the function of the gene product.

*FTO rs9939609*

Table 2 showed the FTO rs9939609 genotype and allele frequencies. The FTO genotypes were in Hardy-Weinberg equilibrium (p = 0.105). The frequencies mutant homozygote genotype (TT) in PTC case and healthy subjects were obtained as 20.0 and 18.7%, respectively. Moreover, the heterozygote genotype (AT) frequency in the case and healthy subjects was 38.8 and 43.7%, respectively. There were no significant differences in the mutant homozygote genotype (TT) and heterozygote genotype (AT) frequencies between PTC cases and controls. Regarding the frequency of mutant allele (T), it was estimated at 39.4% and 40.7% in the PTC cases and healthy subjects, respectively (OR = 0.949, 95% CI = 0.607-1.485, P = 0.819).

**Table 1:** Genotyping Features of SERPINA5 (exon 3) and FTO rs9939609

SNP-ID	Location	Sequence	Fragments size
SERPINA5	Exon 3	F: 5’-GGGACTTTACCTTTGACCTCTACA-3’	394
		R: 5’-GCCACATAATCATTGATCTGCTTC-3’	
FTO rs9939609	Intron 1	5′- AACTGGCTCTTGAATGAAATAGGATTCAGA-3′	C: 154, 33
		5′- AGAGTAACAGAGACTATCCAAGTGCAGTAC-3′	T: 187

**Table 2.** Distribution of the FTO rs9939609 Polymorphism in PTC Cases and Controls

Polymorphism	PTC (n=80)	Controls (n=80)	Odds Ratio		
			OR	90% CI	P-Value
Genotypes					
AA	33 (41.2%)	30 (37.6%)	Ref.		
AT	31 (38.8%)	35 (43.7%)	0.813	0.433-1.528	0.521
TT	16 (20.0%)	15 (18.7%)	1.083	0.494-2.374	0.558
Alleles					
A	97 (60.6%)	95 (59.3%)	Ref.		
T	63 (39.4%)	65 (40.7%)	0.949	0.607-1.485	0.819

OR, Odds Ratio; CI, Confidence Interval

## Discussion

A previous study revealed that the expression level of SERPINA5 is significantly reduced in advanced-stage of ovarian borderline tumors in comparison with the early-stage cases, indicating that this reduction is linked to more aggressive features of the ovarian borderline tumors (Azadi-Yazdiet al., 2017; Nayyaret al., 2017; Baghestaniet al., 2018; Sayadet al., 2020a; Sayadet al., 2020b). To date, a few studies evaluated the association of SERPINA5 polymorphisms with PTC worldwide. Moreover, there was no study on expression level of SERPINA5 in thyroid cancer, although some studies reported over-expression in SERPINA1 in sporadic and radiation-related thyroid cancers (Brenneret al., 2013; Sobhanet al., 2020). Thus, in the current, we examined the associations of SERPINA5 and p53 polymorphisms with risk of PTC in Iranian patients. Our findings showed that SERPINA5 rs6115 and rs6112 polymorphisms might be a novel susceptibility locus for PTC. In 2013, Brenner et al., (2013) performed a study evaluated to identify genetic markers in immune-related pathways and SERPINA5 rs6115, rs6112, rs6108, rs10139508 polymorphisms in 230 candidate gene regions among 344 PTC cases and 452 controls. Their results revealed that the gene collection was not significantly associated with risk of PTC in overall. However, they reported that SERPINA5 rs6115 and rs6112 polymorphisms were significantly associated with PTC at SNPs level. Similarly, they illustrated that these polymorphism are novel susceptibility locus for PTC among US-American patients. In 2016, Sigurdson et al., (2016) evaluated the association of 17525 tag SNPs in 1129 candidate genes with PTC and follicular thyroid cancer (FTC) risk in Germany. Their results revealed several new associations between polymorphisms at SERPINA5, FTO, TICAM1 and HSPA6 genes with PTC risk. Lee et al., (2003) in a study evaluated expression of three genes and DNA methylation using 76 PTC tissues and three thyroid cancer cell lines (TPC1, WRO82-1 and XTC). In their study, SERPINA5 promoter methylation was observed in 7/10 of PTC tissues, while all normal thyroid tissues were un-methylated. Their findings showed that the expression of the MAPK signal-inhibiting gene SERPINA5 decreased in the TPC1 cell line, and SERPINA5 expression was regulated by DNA methylation, which was associated with a higher BRAF mutation rate in PTC (Lee et al., 2013).

The FTO gene has a strong linkage disequilibrium block, within which polymorphisms have been identified that is involved in the development of obesity. Recently some of variants at FTO gene have also been associated with development of cancer (Jafari-Nedooshan et al., 2017). There was no unanimous theory to annotate that obesity related genes can lead to TC. Given the essential role of FTO in development of some cancers and drug resistance, we evaluated the association with risk of PTC in Iranian patients. To the best knowledge, this was the first study to evaluate the possible role of FTO polymorphisms with risk of PTC in Iranian population. Our finding revealed that polymorphism at FTO gene was not associated with risk PTC. In 2012, Kitahara et al., (2012) in a study tested 575 tag SNPs in 23 obesity-related gene regions among 341 incident PTC cases and 444 healthy subjects of European ancestry. Similarly, their results showed that FTO polymorphisms were not associated with susceptibility with PTC. Zhao et al., (2016) in meta-analysis based on 8 studies with 21,810 cases and 85,070 controls evaluated the association of FTOrs8050136 polymorphism with cancer risk. Their pooled data revealed that the FTOrs8050136 polymorphism may be associated with PTC. However, the polymorphism was not associated with pancreatic cancer, endometrial cancer, prostate cancer, colorectal cancer (CRC) and melanoma. However, the meta-analysis relatively small size of participants dwarfed its statistical power. Figlioli et al., (2016) in a meta-analysis combined the results from their genome-wide association study (GWAS) and from published studies on DTC. Their pooled data showed that five polymorphisms including CYP1A1 rs1799814, FTO rs1121980, and the GWAS identified SNPs on 9q22 (rs965513, rs7048394, and rs894673), were statistically associated with DTC after the application of false discovery rate (FDR) correction. 

Considering all the results, this study revealed that SERPINA5 rs6115 and rs6112 polymorphisms might be a novel susceptibility locus for PTC in Iranian patients. However, our findings do not support an association between FTO polymorphism and PTC risk in our population.

## Author Contribution Statement

Conceived and designed the study and experiments: Seyed Mohammad Moshtaghioun, Nasim Fazel-Yazdi, Mohammad Mandegari, Masoud Rahmanian. Performed the experiments: Nasim Fazel-Yazdi, Fatemeh Asadian. Analyzed the data: Nasim Fazel-Yazdi, Fatemeh Asadian, Habib Fazel-Yazdi. All authors have written and approved the manuscript.

## Funding

This study was supported by Yazd University, Yazd, Iran.

## Ethics approval

This study was approved by Yazd University, Yazd, Iran.

## Availability of data and material

 The datasets generated during and/or analyzed during this study are the corresponding author on reasonable request.

## Conflicts of interest

The authors declare that they have no conflict of interest.
